# Supplementary material for: Diagnostic yield and the number of tumor cells of ultrathin bronchoscopy for peripheral lung lesions: A comparison with thin bronchoscopy
Source: PLoS One. 2023 Aug 24;18(8):e0290609. doi: 10.1371/journal.pone.0290609 (PMC10449145; doi:10.1371/journal.pone.0290609)
Supplement: S1 Table — (DOCX) [file pone.0290609.s002.docx]

**S1 Table. Characteristics of 175 patients and lesions.**

|  | Ultrathin bronchoscopy  (n = 69) | Thin bronchoscopy  (n = 106) | p-value |
| --- | --- | --- | --- |
|  | n (%) | n (%) |  |
| Age (median; range) | 71 (36-86) | 72 (34-86) | 0.464 |
| Sex (male) | 56 (81.2) | 75 (70.8) | 0.154 |
| Lesion size (mm, median; range) | 25.0 (6-53) | 26.5 (9-104) | <0.010 |
| Lobar location |  |  | 0.569 |
| Right upper lobe | 19 (27.5) | 30 (28.3) |  |
| Right middle lobe | 6 (8.7) | 8 (7.5) |  |
| Right lower lobe | 16 (23.2) | 18 (17.0) |  |
| Left upper lobe | 15 (21.7) | 34 (32.1) |  |
| Left lower lobe | 13 (18.8) | 16 (15.1) |  |
| Lesion location from the hilum on CT images |  |  | <0.010 |
| Inner | 5 (7.2) | 18 (17.0) |  |
| Middle | 12 (17.4) | 37 (34.9) |  |
| Outer | 52 (75.4) | 51 (48.1) |  |
| Bronchus sign |  |  | <0.010 |
| Present | 67 (97.1) | 79 (74.5) |  |
| Absent | 2 (2.9) | 27 (25.5) |  |
| Characteristics |  |  | <0.010 |
| Solid | 53 (76.8) | 100 (94.3) |  |
| Part solid | 13 (18.8) | 6 (5.7) |  |
| GGN | 3 (4.3) | 0 (0) |  |
| Bronchus generation on VBN  (mean±SD) | 5.2±1.4 | 4.6±1.4 | <0.010 |
| Visible bronchus generation  (mean±SD) | 4.4±1.2 | 3.8±1.0 | <0.010 |
