# Supplementary material for: Diagnostic yield and the number of tumor cells of ultrathin bronchoscopy for peripheral lung lesions: A comparison with thin bronchoscopy
Source: PLoS One. 2023 Aug 24;18(8):e0290609. doi: 10.1371/journal.pone.0290609 (PMC10449145; doi:10.1371/journal.pone.0290609)
Supplement: Attachment — Submitted filename: Response to Reviewers_20230723.docx [file pone.0290609.s004.docx]

Review Comments to the Author

Reviewer #1: A study designed with an effort to reach and diagnose peripheral lung nodules

There are serious methodology errors in the study.

Low number of patients

It is mentioned that the lesions are peripheral, but there is no classification for this.

Which lesion and how much peripheral is unclear?

There was no information on how far the lungs could go with the ultra-thin bronchoscope.

Results and comparison groups are unclear

It is not appropriate for the study to be published in a SCI journal as it is.

**Response)** We are grateful for the time and effort of the reviewer in helping us improve the quality of our manuscript. Your point is valid. Please see our point-by-point responses to the issues raised, as follows. All corrected areas to your comments are indicated by underlines. We hope our answers are satisfactory for you.

Low number of patients

**Response)** Thank you for your suggestion. The number of cases was limited because this was a retrospective study of cases in which ultrathin bronchoscopes were used. Although we mentioned in the limitation that the number of cases is small (P19, L436–P20, L483), we believe that this is a valuable report in that the tumor cell counts were done for both ultrathin and thin bronchoscopy.

We stated that “Fourth, this study was conducted at a single institution and had a small sample size”.

It is mentioned that the lesions are peripheral, but there is no classification for this.

**Response)** Thank you for your suggestion. We defined peripheral as the lesion being invisible by bronchoscopy (P5, L155–6).

We stated that “Peripheral lung lesions were defined as those not visible on bronchoscopy”.

Which lesion and how much peripheral is unclear?

**Response)** Thank you for your comments. We evaluated how much peripheral the lesion was located by bronchus generation on VBN. We also modified the wording of the lesion location with reference to the literature [11]. Specifically, we assessed the lesion location from the hilum on CT images classified into three groups: “inner” for lesions in the inner third ellipses, “middle” for lesions in the middle third ellipses, and “outer” for lesions in the outer third ellipse, and “middle” for lesions. We described lesion location from the hilum on CT images in Table 4.

**Revised version)** P6, L174–7

The lesion location from the hilum on CT images was classified into three groups: “inner” for lesions in the inner third ellipses, “middle” for lesions in the middle third ellipses, and “outer” for lesions in the outer third ellipse, and “middle” for lesions [11].

**Original version)**

Lesion location from the hilum

Central

Intermediate

Peripheral

**Revised version)**

P16 (in Table 4)

Lesion location from the hilum on CT images

Inner

Middle

Outer

There was no information on how far the lungs could go with the ultra-thin bronchoscope.

**Response)** Thank you for correction. Your point is valid. In the present study, the mean bronchus generation reached with the ultrathin bronchoscope was greater than that with the thin bronchoscope (mean±SD, 4.4±1.2 vs. 3.8±1.0, p<0.010). We stated the visible range of the ultrathin bronchoscope in the previous report and added the fact that we were able to observe to a similar extent in our study in the discussion part. We also mentioned in discussion part that the number of tumor cells and diagnostic yield were similar between ultrathin and thin bronchoscopy, despite the fact that ultrathin bronchoscopy was selected for smaller tumors with a greater number of bronchi.

**Revised version)** P19, L419–20

Thus, we were able to reach similar peripheral lesions as previously reported.

**Original version)**

Our study showed that the diagnostic yield and number of tumor cells were similar between ultrathin and thin bronchoscopy, even for smaller tumors with a greater number of bronchi.

**Revised version)** P16, L347-9

Our study showed that the number of tumor cells and diagnostic yield were similar between ultrathin and thin bronchoscopy, despite the fact that ultrathin bronchoscopy was selected for smaller tumors with a greater number of bronchi.

Results and comparison groups are unclear

It is not appropriate for the study to be published in a SCI journal as it is.

**Response)** Thank you for your suggestion. We evaluated the number of tumor cells as the primary endpoint in samples obtained from 37 patients who were histologically diagnosed with NSCLC using ultrathin bronchoscopy and 63 patients using thin bronchoscopy. For clarity, we switched the order of the number of tumor cells part and diagnostic yield part (P12, L279–P14, L304 / P14, L306–P16, L333).

We made efforts to improve this paper by correcting the points raised in this issue.

---

Reviewer #2: The manuscript of Yatani and colleagues aims to compare the diagnostic yield and number of tumor cells of ultrathin bronchoscopy vs. thin bronchoscopy for peripheral lung lesions. With the recent advent and promises of ultrathin bronchoscopy, the results of the current study are of interest for pulmonologists. The current manuscript is well written in a synthetic manner. The study is limited by its retrospective nature.

I would have the following comments:

**Response)** We are grateful for the time and effort of the reviewer in helping us improve the quality of our manuscript. Please see our point-by-point responses to the issues raised, as follows. All corrected areas to your comments are indicated by underlines. We hope our answers are satisfactory for you.

1. The diagnostic yield is the primary endpoint of the study and should be defined more precisely.

**Response)** Thank you very much for important suggestions. Your point is valid. We apologize for the lack of clarity in the primary endpoint. The primary endpoint in this study is the number of tumor cells. For clarity, we switched the order of the number of tumor cells part and diagnostic yield part (P12, L279–P14, L304 / P14, L306–P16, L333).

We also added the exact definition of the diagnostic yield and deleted the diagnostic yield line in Table 4 (original Table 3). Instead, we stated the contents in the text.

We set the cell count as the primary endpoint for this study. We moved to the supporting information part the original Table 2 of characteristics of 175 patients and lesions. Moreover, in the discussion part, we described the content regarding tumor cells first, followed by the content regarding diagnostic yield and the bronchus generation reached by bronchoscopy.

**Revised version)** P14, L307–9

The diagnostic yield was defined as the percentage of cases in which a tissue biopsy was attempted from the lesion by bronchoscopy and some definitive tissue or cellular diagnosis was obtained.

**Original version)**

There were no significant differences in the overall diagnostic yield or the diagnostic yield in lung cancer cases.

**Revised version)** P15, L331

There were no significant differences in the overall diagnostic yield or the diagnostic yield in lung cancer cases (78.6% vs. 78.5%, p=1.000).

2. The current study is retrospective. Did the author try to adjust/compensate for potential selection biases? Only crude statistical comparisons are reported in the Results section. Multivariate statistical analyses could be used.

**Response)** Thank you very much for important suggestions. We performed a multivariate analysis and appended the results and made Table 3 newly. We added its similar content in discussion part. We also added the statistical analysis methods we used in this study in data analyses part.

**Revised version)** P13, L294–9

We performed a multiple regression analysis with the number of tumor cells as the objective variable and bronchoscope selection, lesion size, bronchus generation on VBN, and histological diagnosis as explanatory variables and found that bronchus generation on VBN which meant how peripherally located the lesion was and histological diagnosis could affect the number of tumor cells even when corrected for bronchoscope selection and lesion size. Table 3 shows the result.

P10, L248–P11, L251

We performed the Fisher’s exact tests for qualitative data and t-tests for quantitative data. We also performed multiple regression analysis to evaluate factors affecting the number of tumor cells as the objective variable. We selected items considered clinically relevant and important as explanatory variables.

3. Please report the p-values with at least 3 digits.

**Response)** Thank you very much for important suggestions. We corrected the points noted.

4. Line 219, what does the symbol Â stand for? A table of abbreviations would be useful.

**Response)** Thank you for your comments. This was a particularly meaningless and erroneous statement and removed. Our apologies.

5. Table 3, first line, one should read "Diagnostic yield" instead of Diagnosis yield.

**Response)** Thank you for your comments. We corrected the point noted.

6. If Figure 1 and 2 are independent, Figure 2 left and right panels should be read A and B (instead of C and D).

**Response)** Thank you very much for important suggestions. We corrected the points noted.

---

Reviewer #3: 1. The PI and team can provide additional analysis by also using ANOVA for comparisons of the bronchoscopy. If the assumptions of ANOVA are not meant, The PI and team can go ahead and use non parametrics, especially the Kruskal-Wallis test which compares the median. The team can even use Welch's ANOVA which adjusts for unequal variances and provides reliable results even when the assumption is not met.

**Response)** Thank you very much for important suggestions. We performed a multivariate analysis in order to examine factors affecting the primary endpoint and appended the results and made Table 3 newly.

**Revised version)** P13, L294–9

We performed a multiple regression analysis with the number of tumor cells as the objective variable and bronchoscope selection, lesion size, bronchus generation on VBN, and histological diagnosis as explanatory variables and found that bronchus generation on VBN which meant how peripherally located the lesion was and histological diagnosis could affect the number of tumor cells even when corrected for bronchoscope selection and lesion size. Table 3 shows the result.

2. If possible, the PI and team can cite current literature (> 10years) and remove the old literature as seen in references, page 22 to page 23 lines 369-370, line 372-374, and line 380-381.

**Response)** Thank you very much for important suggestions. As for the first two literature you pointed out, we replaced them with current literature to explain the definitions of the location from the hilum and bronchus sign, respectively, and as for the third paper, we removed it.

**Original version)**

The location from the hilum was classified as central, intermediate, or peripheral. Lesions in the inner third area were classified as central, those in the middle third area as intermediate, and those in the outer third area as peripheral, as classified by Baaklini et al. [11]. Bronchus sign was defined as the presence of bronchi reaching the lesion on thin-slice CT images [12].

**Revised version)** P6, L174–P7, L179

The lesion location from the hilum on CT images was classified into three groups: “inner” for lesions in the inner third ellipses, “middle” for lesions in the middle third ellipses, and “outer” for lesions in the outer third ellipse, and “middle” for lesions [11]. Bronchus sign was defined as the finding on cross-section of a bronchus leading directly to or contained within a nodule or mass [12].

**Original version)**

11. Baaklini WA, Reinoso MA, Gorin AB, Sharafkaneh A, Manian P. Diagnostic yield of fiberoptic bronchoscopy in evaluating solitary pulmonary nodules. Chest. 1991; 117: 1049–54.

12. Gaeta M, Pandolfo I, Volta S, Russi EG, Bartiromo G, Girone G, et al. Bronchus sign on CT in peripheral carcinoma of the lung: value in predicting results of transbronchial biopsy. AJR Am J Roentgenol. 1991; 157: 1181–5.

**Revised version)** P22, L523–30

11. Matsumoto Y, Izumo T, Sasada S, Tsuchida T, Ohe Y. Diagnostic utility of endobronchial ultrasound with a guide sheath under the computed tomography workstation (ziostation) for small peripheral pulmonary lesions. Clin. Respir. J. 2017; 11: 185–92.

12. Guvenc C, Yserbyt, J, Testelmans D, Zanca F, Carbonez A, Ninane V, De Wever W, Dooms C. Computed tomography characteristics predictive for radial EBUS-miniprobe-guided diagnosis of pulmonary lesions. J. Thorac. Oncol. 2015; 10: 472–8.
